# Kindergarten teachers’ emotional intelligence and surface acting: the chain mediating effects of self-efficacy and work engagement

**DOI:** 10.3389/fpsyg.2025.1434407

**Published:** 2025-01-27

**Authors:** Shucun Sun, Zhonglian Yan, Caixia Sun

**Affiliations:** Faculty of Education, Northeast Normal University, Changchun, China

**Keywords:** kindergarten teachers, emotional intelligence, surface acting, self-efficacy, work engagement

## Abstract

**Background:**

Emotional labor, distinct from physical and mental labor, has gained significant attention in contemporary organizational psychology research. As a strategy for performing emotional labor, surface acting refers to the process of faking emotions to meet the demands of organizations’ norms. This study aimed to investigate the relationship between emotional intelligence and surface acting among Chinese kindergarten teachers, focusing on the mediating role of self-efficacy and work engagement.

**Methods:**

Integrating the conservation of resources theory and the job demands-resources model, a questionnaire survey was conducted among 1,017 Chinese kindergarten teachers using Wong & Law Emotional Intelligence Scale, Self-Efficacy Scale, Utrecht Work Engagement Scale, and Surface Acting Scale. SPSS 26.0 was used to conduct descriptive statistics and correlation tests. Structural equation modeling and mediation analysis were constructed using AMOS 28.0.

**Results:**

The results showed reveals several key findings. Firstly, kindergarten teachers’ emotional intelligence significantly positively predicted surface acting rather than negatively. Secondly, self-efficacy and work engagement, respectively, mediate the relationship between emotional intelligence and surface acting in kindergarten teachers. Additionally, self-efficacy and work engagement have chain mediating effects in the relationship between kindergarten teachers’ emotional intelligence and surface acting.

**Conclusion:**

The research findings reveal the influencing mechanism of kindergarten teachers’ emotional intelligence on surface acting, providing a theoretical basis and practical implications for understanding and promoting the reasonable use of surface acting by kindergarten teachers in the Chinese context.

## 1 Introduction

Emotional labor refers to the process by which individuals manage emotions according to the requirements of organizations or work, including natural behavior, deep acting, and surface acting (Hochschild, 1983). In contrast to deep acting, which involves individuals’ expressing their true emotions, surface acting refers to the sole modification of external expression without altering internal emotions (Diefendorff et al., 2005). This concept aligns with the response-focused strategy in the emotion regulation model (Grandey, 2000). According to the job demands-resources (JD-R) model, the performance rules for emotional expressions is classified as a job demand, which can contribute to work pressure. Consequently, surface acting plays an essential role in both individuals’ psychological outcomes and organizational performances (Bakker et al., 2023; Li et al., 2024a,b).

Kindergarten teaching is a profession that requires substantial emotional labor (Näring et al., 2006). This role involves not only instructional tasks but also non-instructional duties and daily child care (Hargreaves, 2000). For instance, they must collaborate with colleagues to manage teaching and care responsibilities, or communicate regularly with parents regarding the children’s performance. As a result, kindergarten teachers need a stronger emotional modification, leading to an increased surface-acting frequency. Some studies believe that surface acting may increase resignation intentions, reduce sleep quality (Gu et al., 2020), increase work–family conflict, reduce happiness (Gu and Wang, 2023), increase emotional exhaustion, and reduce teaching satisfaction (Yin et al., 2019). These studies highlight the need for interventions to reduce surface acting among kindergarten teachers, as doing so can both improve the quality of teaching and care work, and enhance interactions between teachers and children (Lim et al., 2024). There are also studies indicating that higher surface acting may have positive effects, increasing employee dedication (Lee and Madera, 2019), and improving individuals’ health levels (Lennard et al., 2019).

Therefore, how to correctly understand and optimize surface acting has become an urgent issue in both the academic and practical fields. In the causal model of emotional labor (Grandey, 2000), emotional intelligence as a personal factor has an important impact on surface acting, but the specific underlying mechanism has not been explored (Law et al., 2004). Current research mostly regards surface acting as a process variable between antecedent and outcome variables (Huang and Yin, 2024), focusing solely on the direct effect of emotional intelligence on surface acting (Yin et al., 2013; Lee and Chelladurai, 2015). However, there is limited exploration of the underlying mediation mechanisms, making it difficult to explain how emotional intelligence influences surface acting. Therefore, it is necessary to further explore the role of influencing factors in surface acting (Rucker et al., 2011).

**Figure 1:**
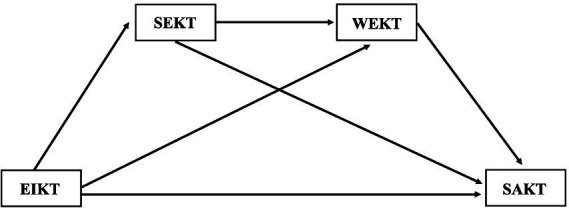
Hypothesized research model. EIKT = emotional intelligence of kindergarten teachers, SEKT = self-efficacy of kindergarten teachers, WEKT = work engagement of kindergarten teachers, SAKT = Surface acting of kindergarten teachers.

This research was conducted on kindergarten teachers in China to explore the influences of emotional intelligence on surface acting based on the conservation of resources (COR) theory and the JD-R model (Bakker et al., 2023). Specifically, we focused on how self-efficacy and work engagement mediate the relationship between emotional intelligence and surface acting. The aim was to uncover the mechanism through which emotional intelligence influences surface acting. This study uses a chain mediating model to examine the relationship between emotional intelligence and surface acting for kindergarten teachers, and proposes the following hypotheses: (1) Emotional intelligence of kindergarten teachers can significantly predict surface acting; (2) Self-efficacy mediates the relationship between emotional intelligence and surface acting; (3) Work engagement mediates the relationship between emotional intelligence and surface acting; (4) Self-efficacy and work engagement have chain mediating effects in the relationship between emotional intelligence and surface acting in kindergarten teachers. This research framework is illustrated in Figure 1. By identifying additional factors contributing to surface acting and extending the theoretical research on surface acting in kindergarten teachers, this study sought to provide practical insights for optimizing surface acting.

## 2 Literature review

### 2.1 Emotional intelligence and surface acting

The exploration of influencing factors of surface acting is an important topic in research on teachers’ emotional labor (Akın et al., 2013; Hong and Zhang, 2019; Zheng et al., 2020). In related studies, the regulation mechanism model of emotional labor is relatively classic and accepted by most scholars (Grandey, 2000). This model points out that there are four types of influencing factors on surface acting, with personal factors being the most prominent (Tang and Gu, 2024). Emotional intelligence, as a core psychological trait of teachers, is one of the most important personal factors affecting surface acting (Yin et al., 2013; Lee and Chelladurai, 2015; Karakus et al., 2024). Emotional intelligence refers to the ability of individuals to “monitor their own and others’ feelings and emotions, distinguish these feelings, and use this information to guide their thinking and actions” (Salovey and Mayer, 1990). Emotional intelligence can help individuals perceive the emotional rules of the work environment and more effectively choose which type of emotional labor to adopt (Kokkinos and Voulgaridou, 2024).

Existing research conclusions on the relationship between teachers’ emotional intelligence and surface acting vary greatly, and the specific impact of emotional intelligence on teachers’ surface acting still needs further verification. A small amount of research has found a positive correlation or no correlation between teachers’ emotional intelligence and surface acting (Prentice et al., 2013; Pervaiz et al., 2019; Yin et al., 2013), but more studies believe that there is a negative correlation between teachers’ emotional intelligence and surface acting (Yin, 2015; Lee and Chelladurai, 2017; Kang, 2020; Karakus et al., 2024). Teachers with good emotional intelligence can adaptively regulate their emotions, attitudes, and behaviors when facing work pressure. Therefore, the emotional intelligence of kindergarten teachers may negatively predict surface acting.

### 2.2 The mediating effect of self-efficacy

Teachers’ self-efficacy originates from Albert Bandura’s social learning theory and is a subjective perception and judgment of teachers’ influence on educational value, their teaching level, and children’s learning and development abilities. It includes teachers’ efficacy perceptions of teaching strategies, classroom management, and student classroom participation (Tschannen Moran et al., 1998). Social cognitive theory suggests that self-perception and self-regulation play a key role in the formation of self-efficacy (Bandura et al., 1999). Emotional intelligence mainly emphasizes individuals’ perception and regulatory ability of their emotional states, which may affect the development of teachers’ self-efficacy. Research suggests that emotional intelligence is significantly positively correlated with teachers’ self-efficacy, and high emotional intelligence can enhance kindergarten teachers’ self-efficacy (Chan, 2004; Aparisi et al., 2020; Lu and Ishak, 2022).

The COR theory suggests that employees with high self-efficacy are more conscientious, tend to exert more effort to achieve work goals, and use surface acting less (Hobfoll, 1989; Janssen, 2004). In other words, the higher the teacher’s self-efficacy, the less likely they are to use surface acting (Schwab, 2019). Self-efficacy can affect teachers’ attitudes, behaviors, and mindset when dealing with various challenges. Burić and Mornar (2023) conducted a longitudinal study, indicating that teachers with low self-efficacy are more likely to use surface acting.

### 2.3 The mediating effect of work engagement

Work engagement refers to having positive behaviors, emotions, and cognitive states in the workplace, which is a positive and fulfilling mental state related to work. It typically includes three dimensions: vigor, dedication, and absorption (Schaufeli et al., 2002). The level of work engagement of kindergarten teachers directly affects the quality of care and the physical and mental health of children, making it crucial to enhance the level of work engagement of kindergarten teachers (Turin and Davidson, 2022). According to the JD-R model, factors influencing work can be divided into job demands and job resources (Bakker et al., 2023). Emotional intelligence is the ability to control emotions, which belongs to job resources. Teachers with high emotional intelligence can better understand and control their emotions, handle challenges and pressures in their work more effectively, and have higher work engagement. The emotional intelligence of kindergarten teachers has a significant impact on work engagement (Chakraborty and Saha., 2022).

Based on the COR theory, individuals consume a large amount of emotional resources when performing emotional labor (Hobfoll, 1989). If the acquisition of resources cannot balance the loss of resources, employees’ attitudes and performance will be negatively affected. Employees with high levels of work engagement can wholeheartedly immerse themselves in their work, possessing more individual resources and intrinsic motivation to display the emotions required by organizations (Liu et al., 2008). Surface acting often brings negative impacts to individuals and organizations, reflecting a relatively low professional identity of individuals. Therefore, individuals with higher levels of work engagement may use surface acting less (Hülsheger and Schewe, 2011). High levels of work engagement indicate high energy and strong identification with work, suggesting that individuals are focused and not distracted (Schaufeli et al., 2006). Existing research has indicated that work engagement promotes employees’ task performance, while surface acting often falls into the category of task performance with negative effects (Karatepe, 2013). The higher the individual’s work engagement, the more willing the employee is to achieve high job performance and the less likely they are to use surface acting (Zvobgo et al., 2021). Gabriel et al. (2015) also found similar results, where employees with positive emotions tend not to use surface acting, while those with negative emotions tend to use surface acting.

### 2.4 The chain mediation effects of teachers’ self-efficacy and work engagement

Self-determination theory provides insights into the relationship between emotional intelligence and surface acting for kindergarten teachers. This theory suggests that individuals have three basic psychological needs: autonomy, competence, and relatedness. When these needs are met, it enhances work motivation and willingness (Baard et al., 2004), and further promotes other positive behavioral outcomes, such as inhibiting surface acting (Phuoc et al., 2022). Individuals with high emotional intelligence are more likely to recognize the need to frequently incorporate emotions as part of their work role, and thus choose to adopt or not adopt surface acting based on situational demands. The improvement of emotional intelligence among kindergarten teachers contributes to an enhanced sense of control over the work environment, activates proactive concern for children’s needs, and fosters a caring interpersonal atmosphere to meet competence, autonomy, and other psychological needs (Cameron, 2022). This can enhance motivation, increase self-efficacy, work engagement, and inhibit surface acting (Christie et al., 2007). According to social cognitive theory, self-efficacy is an important foundation for action, and individuals will only take action if they believe their actions can achieve the expected results (Adebusuyi et al., 2022). Therefore, the higher the teacher’s self-efficacy, the more likely they are to maintain a positive work state and be highly engaged in their work (Kim, 2023). Teachers’ self-efficacy significantly positively predicts work engagement (Tan and Chou, 2018).

## 3 Methods

### 3.1 Participants

This study selected kindergarten teachers from eastern provinces with highly developed economies, including Jiangsu, Shandong, and Hebei, as well as moderately developed central provinces, such as Hunan, Shanxi, and Hubei, as participants. An online questionnaire was distributed to collect data from these regions. The samples were drawn from regions with varying levels of economic development, aiming to capture the characteristics of kindergarten teachers from both highly and moderately developed areas. A total of 1,051 questionnaires were collected, 34 invalid questionnaires were excluded, resulting in 1,017 valid questionnaires, with an effective rate of 96.8%. The sample distribution by province was as follows: 173 respondents from Jiangsu (17.0%), 152 from Shandong (14.9%), 183 from Hebei (18.0%), 156 from Hunan (15.3%), 168 from Shanxi (16.5%), and 185 from Hubei (18.2%); 96.0% were female teachers, and 4.0% were male teachers. 73.5% were aged thirty or younger, 15.9% were in their thirties, 10.6% were over forty years old. Regarding teaching experience, 58.5% had been teaching for three years or less, 17.5% had between four and six years of experience, 6.9% had between seven and nine years of experience, and 17.1% had more than nine years of teaching experience. 41.3% were married, and 58.7% were unmarried. 33.2% had official positions, and 66.8% did not.

### 3.2 Procedure

Before conducting the research, ethical approval was obtained from the Ethics Committee of the author’s university. Prior to the distribution of the questionnaires, participating kindergarten teachers were informed of the research objectives and content. Participation in the study was voluntary, with all participants providing informed consent. The gathered information was kept anonymous and confidential, in accordance with the ethical principles outlined in the Declaration of Helsinki. Kindergarten teachers independently filled out the questionnaire according to their actual situations, and the filling process took about 15 min. Finally, the model was established based on analyzed statistics.

### 3.3 Research tools

#### 3.3.1 Utrecht Work Engagement Scale

The Utrecht Work Engagement Scale (UWES), developed by Schaufeli et al. (2002), was used in this study. The scale consists of three dimensions: vitality, dedication, and absorption, rated on a 7-point scale ranging from “never” (0 points) to “always” (6 points), with a total of 9 items. An example item is “I am enthusiastic about my work.” A higher score indicates a higher level of work engagement. The Cronbach’s alpha coefficient for this scale was 0.951, and the CFA fit was good (χ^2^/df = 4.000, RMSEA = 0.063, CFI = 0.990, GFI = 0.980, NFI = 0.990, IFI = 0.992).

#### 3.3.2 Wong and Law Emotional Intelligence Scale

The Wong & Law Emotional Intelligence Scale (WLEIS), developed by Wong and Law (2002), was employed in this study. The scale utilizes a 5-point rating scale ranging from “strongly disagree” (1 point) to “strongly agree” (5 points), comprising 16 items distributed across four dimensions: self-emotion appraisal, others’ emotion appraisal, regulation of emotion, and use of emotion. An example item is “I really understand my own feelings.” A higher score indicates a higher level of emotional intelligence. The Cronbach’s alpha coefficient for this scale was 0.937, and the CFA fit was good (χ^2^/df = 4.836, RMSEA = 0.061, CFI = 0.965, GFI = 0.946, NFI = 0.956, IFI = 0.965).

#### 3.3.3 Teacher Self-Efficacy Scale

The Teacher Self-Efficacy Scale (TSES), developed by Tschannen-Moran and Hoy (2001), was used to measure the self-efficacy of kindergarten teachers. It consists of three dimensions: student engagement efficacy, teaching strategy efficacy, and class management efficacy. An example item is “To what extent can you provide an alternative explanation or example when children are confused?” A higher score indicates higher self-efficacy. This scale uses a 9-point rating system, ranging from “completely unable to do” (1 point) to “completely able to do” (9 points), with a total of 12 items. The Cronbach’s alpha coefficient for this scale was 0.960, and the CFA fit was good (χ^2^/df = 4.658, RMSEA = 0.060, CFI = 0.983, GFI = 0.993, NFI = 0.983, IFI = 0.983).

#### 3.3.4 Surface Acting Scale

The Surface Acting Scale (SAS), developed by Diefendorff et al. (2005), was used to measure the surface acting of kindergarten teachers. This scale employs a 5-point rating system, ranging from “strongly disagree” (1 point) to “strongly agree” (5 points), with a total of 7 items, without distinguishing dimensions. An example item is “When facing children, I would hide my true feelings to display appropriate emotions.” A higher score indicates a greater use of surface acting. The Cronbach’s alpha coefficient for this scale was 0.800, and the CFA fit was good (χ^2^/df = 4.880, RMSEA = 0.062, CFI = 0.987, GFI = 0.970, NFI = 0.996, IFI = 0.973).

#### 3.3.5 Statistical analysis

SPSS 26.0 software program was used for method deviation testing, descriptive statistical analysis, and correlation analysis of variables. AMOS 28.0 was employed for testing the structural equation model and Bootstrap analysis. The Bootstrap method was used to extract samples 5,000 times to estimate a 95% confidence interval.

## 4 Results

### 4.1 Common method bias test

All variables in this study were reported by kindergarten teachers. The Harman single-factor test was used to conduct an unrotated factor analysis on all questionnaire items. The exploratory factor analysis without rotation yielded a total of 6 factors with eigenvalues greater than 1. The maximum factor variance explained was 20.26%, which is less than 40%. Therefore, this study does not exhibit significant common method bias (Fuller et al., 2016).

### 4.2 Descriptive statistics and correlation analysis

The results of descriptive statistics and correlation analysis are shown in Table 1, indicating significant correlations between emotional intelligence, self-efficacy, work engagement, and surface acting. Specifically, emotional intelligence is significantly positively correlated with self-efficacy, work engagement, and surface acting, respectively. Self-efficacy is significantly positively correlated with work engagement. Self-efficacy and work engagement are significantly negatively correlated with surface acting, respectively.

**Table 1:** Mean, standard deviation and correlation coefficient matrix for each variable (*N* = 1,017).

	EIKT	SEKT	WEKT	SAKT
EIKT	1			
SEKT	0.645**	1		
WEKT	0.606**	0.673**	1	
SAKT	0.330**	−0.174**	−0.159*	1
M	4.056	7.463	3.952	3.441
SD	0.650	1.199	0.884	0.785

*p < 0.05, **p < 0.01, and ***p < 0.001.

### 4.3 Chain mediating effects

**Figure 2:**
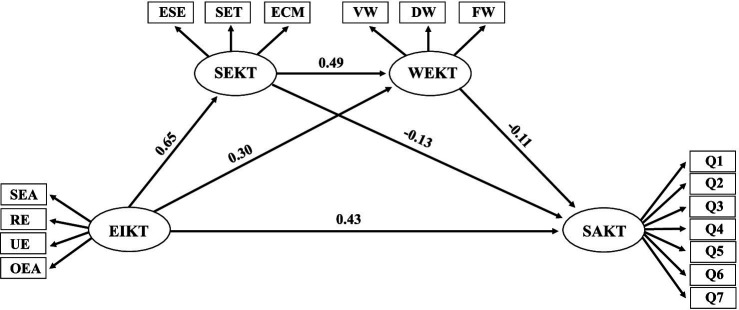
A model of the relationships among emotional intelligence of kindergarten teachers, self-efficacy of kindergarten teachers, work engagement of kindergarten teachers and surface acting of kindergarten teachers (SEA = Self-Emotional Appraisal; RE = Regulation of Emotion; UE = Use of Emotion; OEA = Others’ Emotional Appraisal; ESE = Efficacy of Student Engagement; SET = Efficacy of Teaching Strategy; ECM = Efficacy of Class Management; VW = Vitality at Work; DW = Dedication of Work; FW = Focus on Work).

To further investigate the relationships between emotional intelligence, surface acting, self-efficacy, and work engagement among kindergarten teachers, and to test the mediating effects of self-efficacy and work engagement, a latent variable structural equation model was constructed to establish a relationship model between the four variables, as shown in Figure 2. The results show that the fit indices of the model are: χ^2^/df = 7.046, RMSEA = 0.077, NFI = 0.930, IFI = 0.940, GFI = 0.930, CFI = 0.912. All fit indices are good, indicating a good fit between the data and the constructed model (Yan et al., 2023).

Analysis of the paths in this structural equation model reveals that emotional intelligence has a significantly positive predictive effect on surface acting (*β* = 0.43, *p* < 0.001), supporting hypothesis 1. Emotional intelligence also has a significantly positive predictive effect on self-efficacy (*β* = 0.65, *p* < 0.001), self-efficacy has a significantly positive predictive effect on work engagement (*β* = 0.49, *p* < 0.001), and work engagement has a significantly negative predictive effect on surface acting (*β* = −0.11, *p* < 0.01). Emotional intelligence has a significantly positive predictive effect on work engagement (*β* = 0.30, *p* < 0.001), and self-efficacy has a significantly negative predictive effect on surface acting (*β* = −0.13, *p* < 0.01).

The total mediating effect values of self-efficacy and work engagement in the relations between emotional intelligence and surface acting is −0.170. Specifically, the mediation effect of emotional intelligence on surface acting consists of three indirect effect values generated by three paths: (1) the indirect effect value generated by the path of emotional intelligence → self-efficacy → surface acting is −0.093; (2) the indirect effect value generated by the path of emotional intelligence → work engagement → surface acting is −0.038; and (3) the indirect effect value generated by the path of emotional intelligence → self-efficacy → work engagement → surface acting is −0.039.

Bootstrap analysis was conducted with a confidence interval set at 95%. If the 95% confidence interval of the mediation effect does not include 0, it indicates that the mediation effect is significant. The 95% confidence intervals of each path from emotional intelligence to surface acting are shown in Table 2. From the results in Table 2, it can be observed that the confidence intervals for the mediation effect values generated by the path of emotional intelligence → self-efficacy → surface acting, the path of emotional intelligence → work engagement → surface acting, and the path of emotional intelligence → self-efficacy → work engagement → surface acting do not include 0, indicating that all three indirect effects reach significance, thus hypotheses 2, 3 and 4 are both supported.

**Table 2:** Effect values and confidence intervals for the mediating effects analysis.

Path relationship	Estimated	95% confidence interval	
		Upper limit	Lower limit
Total mediation effect	−0.170	−0.091	−0.260
EIKT → SEKT → SAKT	−0.093	−0.014	−0.188
EIKT → WEKT → SAKT	−0.038	−0.004	−0.082
EIKT → SEKT → WEKT → SAKT	−0.039	−0.003	−0.077

Bootstrap size = 5,000. CI = confidence interval.

## 5 Discussion

This study utilized a chain mediating model to elucidate the influence of emotional intelligence on surface acting in kindergarten teachers. The results indicate a positive correlation between emotional intelligence and surface acting among kindergarten teachers, and self-efficacy and work engagement play a simple chain mediating role between emotional intelligence and surface acting.

### 5.1 Positive correlation between emotional intelligence and surface acting

The research results indicate a significant impact of emotional intelligence on surface acting among kindergarten teachers. The study demonstrates that emotional intelligence positively, rather than negatively, predicts surface acting. In other words, the higher the emotional intelligence of kindergarten teachers, the higher their levels of surface acting. This finding contradicts the results of most studies, including those by Yin (2012), Lee and Chelladurai (2017), Kang (2020), and Karakus et al. (2024), but is consistent with the findings of other research, such as those of Karim and Weisz (2011), Prentice et al. (2013), and Pervaiz et al. (2019). Considering the nature and characteristics of kindergarten teachers’ work, there are two possible explanations. One is that kindergarten teachers work long hours with frequent emotional interactions, making them high emotional laborers (Qi et al., 2017). The high level of emotional labor weakens the ability of kindergarten teachers to effectively regulate emotions, leading to an exacerbation of surface acting (Glomb and Tews, 2004). Wang et al. (2019) also suggested that kindergarten teachers with high emotional intelligence tend to engage in surface acting to cope with high emotional consumption. Another explanation is that kindergarten teachers deliberately use surface acting to achieve teaching objectives. The unique characteristic of kindergarten teaching tasks requires teachers to maintain intimate interactions with young children through surface acting, thus fostering strong bonds with the children and establishing a family-like relationship (Li et al., 2024a,b; Xu et al., 2024). Yin and Lee (2012) pointed out that teachers use surface acting as a means to achieve teaching goals. Experienced teachers are good at employing exaggerated facial expressions and body movements to attract the attention of young children. The higher the emotional intelligence of kindergarten teachers, the more likely they may engage in surface acting (Gutiérrez Cobo et al., 2017). In this context, deliberate surface acting reflects affection toward young children and illustrates the care ethics upheld by Chinese kindergarten teachers (Xu et al., 2024).

### 5.2 The mediating role of kindergarten teachers’ self-efficacy

This study demonstrates that the self-efficacy of kindergarten teachers mediates the relationship between emotional intelligence and surface acting, supporting hypothesis 2. Emotional intelligence is significantly positively correlated with self-efficacy, consistent with the findings of research (Chan, 2004; Aparisi et al., 2020). According to emotional intelligence theory, emotional intelligence involves individual self-perception and self-regulation, which play a crucial role in the development of self-efficacy. Individuals with high emotional intelligence have a better understanding of themselves, which increases their confidence in handling interpersonal relationships and tasks, leading to higher self-efficacy (Udayar et al., 2020). The self-efficacy of kindergarten teachers negatively predicts surface acting, consistent with the findings of research (Schwab, 2019; Burić and Mornar, 2023). Self-efficacy serves as an effective feedback mechanism for teachers facing work pressure and conflicts, determining their stress response (Burić and Mornar, 2023). Teachers with high self-efficacy can better adjust their mood and behavior, actively deal with challenges at work, and reduce surface acting.

### 5.3 The mediating role of kindergarten teachers’ work engagement

The work engagement of kindergarten teachers mediates the relationship between emotional intelligence and surface acting, thus verifying hypothesis 3. Emotional intelligence is significantly positively correlated with work engagement, aligning with the findings of Chakraborty and Saha (2022). As a personal psychological resource and protective factor, emotional intelligence can increase beneficial behaviors (Willoughby and Boutwell, 2018; Jung et al., 2019; Kang, 2020). As an ability to control emotions, it helps individuals alleviate the fatigue of engagement caused by insufficient resources and enhances work engagement (Mérida López and Extremera, 2020). The work engagement of kindergarten teachers negatively predicts surface acting, consistent with the findings of research (Karatepe, 2013; Zvobgo et al., 2021; Burić and Mornar, 2023). This may be because surface acting often leads to negative effects (Sousan et al., 2022; Sciotto and Pace, 2022). In order to achieve better educational outcomes, kindergarten teachers with high work engagement actively reduce surface acting to improve their teaching and care work (Tiwari and Shukla, 2023).

### 5.4 The chain mediating effects of kindergarten teachers’ self-efficacy and work engagement

This study indicates that the self-efficacy and work engagement of kindergarten teachers have chain mediating effects in the relationship between emotional intelligence and surface acting, supporting hypothesis 4. This indicates that the emotional intelligence of kindergarten teachers not only affects surface acting through the separate mediation effects of self-efficacy and work engagement but also influences work engagement through self-efficacy, thereby affecting surface acting. The significant positive prediction of self-efficacy of kindergarten teachers on work engagement is consistent with previous research findings (Chan et al., 2017). According to the self-efficacy theory (Bandura et al., 1980), individuals assess their abilities based on emotional states. If kindergarten teachers can manage emotional issues well, they will experience more positive emotions psychologically and behaviorally, leading to higher self-efficacy in activities such as classroom management and teacher-child interactions, thus better adjusting their mood and behavior, increasing work engagement, and reducing surface acting (Abbasi et al., 2021). The above analysis also validates the viewpoint of the “protective factor-protective factor” model, wherein one protective factor can enhance the effects of another protective factor. Emotional intelligence, as a protective factor for the emotional labor of kindergarten teachers, can strengthen the effects of self-efficacy and work engagement, which are also protective factors (McCray and Joseph Richard, 2020). At the same time, the results of this study further explain the motivational process of the JD-R model. As a “positive job resource,” emotional intelligence can facilitate individual development, serving as a valuable personal resource that meets the demands of teaching and care work in kindergartens (Turner and Stough, 2020). Additionally, self-efficacy, as a psychological capital, is also integrated into the JD-R model. The motivational process illustrates that high levels of self-efficacy can lead to increased motivation, thereby improving teacher’s work engagement (Bakker et al., 2023; Li et al., 2024a,b). Therefore, the chain mediation of “self-efficacy → work engagement” is also an important bridge for the influence of emotional intelligence of kindergarten teachers on their surface acting.

### 5.5 Main contributions and limitations

The findings of the current study have theoretical and practical significance. Theoretically, this study largely validates the interaction among influencing factors of surface behavior (Grandey and Melloy, 2017) and explores the underlying mechanism between emotional intelligence and surface acting, extending and further refining the model based on Grandey’s classic causal model of emotional labor, deepening the theoretical explanation of factors influencing surface acting of kindergarten teachers (Grandey, 2000). Practically, this study provides new intervention and support pathways for promoting the rational use of surface acting by kindergarten teachers and enhancing their occupational psychological health. While emphasizing the development of emotional intelligence of kindergarten teachers, various measures should be taken to address the occupational challenges of long working hours and high emotional demands. Educational training should be conducted on the rational use of surface acting to enhance the emotional competence of kindergarten teachers (Levine Brown et al., 2022). Additionally, efforts should be made to enhance the professional competence of kindergarten teachers and strengthen their self-efficacy. As a cognitive motivational mechanism, self-efficacy represents an individual’s belief in their work ability, which significantly influences their work status. Kindergarten teachers should prioritize and consider it as a controllable and developable positive psychological resource (Heo et al., 2022). Lastly, kindergarten teachers should be encouraged to maintain high work engagement. As a positive experience, high work engagement enables teachers to work happily and efficiently, which can optimize surface acting (Zvobgo et al., 2021).

There are still some limitations in this study. First, while the Harman single-factor test method was utilized to confirm the absence of significant common method bias, reliance on self-reported questionnaires from kindergarten teachers may potentially inflate the associations between variables. Therefore, future research could use various methods such as leadership evaluation or peer assessment to collect variables in the study. Second, the cross-sectional nature of the data used in this study can only reflect the correlation between variables but cannot reflect the directionality of the relationships between variables. Future research should use longitudinal data to examine the relationships between variables. Third, the study required kindergarten teachers to recall surface acting in their work, and retrospective assessments may be inaccurate. Future research could incorporate methods such as on-site observations.

## 6 Conclusion

Self-efficacy and work engagement mediate the relationship between emotional intelligence and surface acting in kindergarten teachers, demonstrating both simple and chain mediation effects. These results indicate that self-efficacy and work engagement may play important roles in the relationship between emotional intelligence and surface acting. The findings provide theoretical basis and practical implications for promoting the rational use of surface acting by kindergarten teachers. It is suggested that kindergarten administrators should permit reasonable surface acting to capture the attention of young children when designing emotional display rules.

## Data Availability

The original contributions presented in the study are included in the article/supplementary material, further inquiries can be directed to the corresponding author.

## References

[ref1] Abbasi F. Ghahremani L. Nazari M. Fararouei M. Khoramaki Z. Curcuruto M. (2021). Lifestyle in female teachers: educational intervention based on self-efficacy theory in the south of Fars province, Iran. BioMed Res. Int. 2021, 1–8. doi: 10.1155/2021/6177034, PMID: 34912893 PMC8668293

[ref2] Adebusuyi A. S. Adebusuyi O. F. Kolade O. (2022). Development and validation of sources of entrepreneurial self-efficacy and outcome expectations: A social cognitive career theory perspective. Int. J. Manag. Educ. 20:100572. doi: 10.1016/j.ijme.2021.100572

[ref3] Akın U. Aydın İ. Erdoğan Ç. Demirkasımoğlu N. (2013). Emotional labor and burnout among Turkish primary school teachers. Aust. Educ. Res. 41, 155–169. doi: 10.1007/s13384-013-0138-4

[ref4] Aparisi D. Granados L. Sanmartín R. Martínez Monteagudo M. C. García Fernández J. M. (2020). Relationship between emotional intelligence, generativity and self-efficacy in secondary school teachers. Sustain. For. 12:3950. doi: 10.3390/su12103950

[ref5] Baard P. P. Deci E. L. Ryan R. M. (2004). Intrinsic need satisfaction: A motivational basis of performance and weil-being in two work settings1. J. Appl. Soc. Psychol. 34, 2045–2068. doi: 10.1111/j.1559-1816.2004.tb02690.x

[ref6] Bakker A. B. Demerouti E. Sanz-Vergel A. (2023). Job demands-resources theory: ten years later. Annu. Rev. Organ. Psych. Organ. Behav. 10, 25–53. doi: 10.1146/annurev-orgpsych-120920-053933

[ref7] Bandura A. Adams N. E. Hardy A. B. Howells G. N. (1980). Tests of the generality of self-efficacy theory. Cogn. Ther. Res. 4, 39–66. doi: 10.1007/BF01173354

[ref8] Bandura A. Freeman W. H. Lightsey R. (1999). Self-efficacy: the exercise of control. J. Cogn. Psychother. 13, 158–166. doi: 10.1891/0889-8391.13.2.158

[ref9] Burić I. Mornar M. (2023). Teacher dispositional affectivity, emotional labor, and self-efficacy: A longitudinal analysis. Curr. Psychol. 42, 18382–18395. doi: 10.1007/s12144-022-03029-7

[ref10] Cameron L. D. (2022). “Making out” while driving: relational and efficiency games in the gig economy. Organ. Sci. 33, 231–252. doi: 10.1287/orsc.2021.1547, PMID: 19642375

[ref11] Chakraborty D. Saha S. (2022). Job involvement in relation to emotional intelligence of the heads of higher secondary schools in the West Bengal. Towards Excell. 14, 1445–1459. doi: 10.37867/te1402120

[ref12] Chan D. W. (2004). Perceived emotional intelligence and self-efficacy among Chinese secondary school teachers in Hong Kong. Personal. Individ. Differ. 36, 1781–1795. doi: 10.1016/j.paid.2003.07.007

[ref13] Chan X. W. Kalliath T. Brough P. O’Driscoll M. Siu O. L. Timms C. (2017). Self-efficacy and work engagement: test of a chain model. Int. J. Manpow. 38, 819–834. doi: 10.1108/IJM-11-2015-0189

[ref14] Christie A. Jordan P. Troth A. Lawrence S. (2007). Testing the links between emotional intelligence and motivation. J. Manag. Organ. 13, 212–226. doi: 10.5172/jmo.2007.13.3.212

[ref15] Diefendorff J. M. Croyle M. H. Gosserand R. H. (2005). The dimensionality and antecedents of emotional labor strategies. J. Vocat. Behav. 66, 339–357. doi: 10.1016/j.jvb.2004.02.001

[ref16] Fuller C. M. Simmering M. J. Atinc G. Atinc Y. Babin B. J. (2016). Common methods variance detection in business research. J. Bus. Res. 69, 3192–3198. doi: 10.1016/j.jbusres.2015.12.008

[ref17] Gabriel A. S. Daniels M. A. Diefendorff J. M. Greguras G. J. (2015). Emotional labor actors: A latent profile analysis of emotional labor strategies. J. Appl. Psychol. 100, 863–879. doi: 10.1037/a0037408, PMID: 25068812

[ref18] Glomb T. M. Tews M. J. (2004). Emotional labor: A conceptualization and scale development. J. Vocat. Behav. 64, 1–23. doi: 10.1016/S0001-8791(03)00038-1

[ref19] Grandey A. A. (2000). Emotional regulation in the workplace: A new way to conceptualize emotional labor. J. Occup. Health Psychol. 5, 95–110. doi: 10.1037/1076-8998.5.1.95, PMID: 10658889

[ref20] Grandey A. A. Melloy R. C. (2017). The state of the heart: emotional labor as emotion regulation reviewed and revised. J. Occup. Health Psychol. 22, 407–422. doi: 10.1037/ocp0000067, PMID: 28150996

[ref21] Gu Y. Wang R. (2023). Why and when surface acting interferes with family functioning: the role of psychological detachment and family-supportive supervisor behaviors. Curr. Psychol. 42, 21227–21238. doi: 10.1007/s12144-023-04319-4, PMID: 36742064 PMC9885067

[ref22] Gu Y. You X. Wang R. (2020). Workplace surface acting and employee insomnia: A moderated mediation model of psychological detachment and dispositional mindfulness. J. Psychol. 154, 367–385. doi: 10.1080/00223980.2020.1757595, PMID: 32394806

[ref23] Gutiérrez Cobo M. J. Cabello R. Fernández Berrocal P. (2017). Performance-based ability emotional intelligence benefits working memory capacity during performance on hot tasks. Sci. Rep. 7:11700. doi: 10.1038/s41598-017-12000-7, PMID: 28916754 PMC5600979

[ref24] Hargreaves A. (2000). Mixed emotions: teachers' perceptions of their interactions with students. Teach. Teach. Educ. 16, 811–826. doi: 10.1016/S0742-051X(00)00028-7

[ref25] Heo H. Bonk C. J. Doo M. Y. (2022). Influences of depression, self-efficacy, and resource management on learning engagement in blended learning during COVID-19. Internet High. Educ. 54:100856. doi: 10.1016/j.iheduc.2022.100856, PMID: 35464172 PMC9013013

[ref26] Hobfoll S. E. (1989). Conservation of resources: a new attempt at conceptualizing stress. Am. Psychol. 44, 513–524. doi: 10.1037/0003-066x.44.3.513, PMID: 2648906

[ref27] Hochschild A. R. (1983). The managed heart: Commercialization of human feeling. Berkeley: University of California Press.

[ref28] Hong X. Zhang M. (2019). Early childhood teachers’ emotional labor: a cross-cultural qualitative study in China and Norway. Eur. Early Child. Educ. Res. J. 27, 479–493. doi: 10.1080/1350293x.2019.1634235

[ref29] Huang S. Yin H. (2024). The relationships between paternalistic leadership, teachers’ emotional labor, engagement, and turnover intention: A multilevel SEM analysis. Teach. Teach. Educ. 143:104552. doi: 10.1016/j.tate.2024.104552

[ref30] Hülsheger U. R. Schewe A. F. (2011). On the costs and benefits of emotional labor: A meta-analysis of three decades of research. J. Occup. Health Psychol. 16, 361–389. doi: 10.1037/a0022876, PMID: 21728441

[ref31] Janssen O. (2004). The barrier effect of conflict with superiors in the relationship between employee empowerment and organizational commitment. Work Stress 18, 56–65. doi: 10.1080/02678370410001690466

[ref32] Jung Y. Shin N. Y. Jang J. H. Lee W. J. Lee D. Choi Y.. (2019). Relationships among stress, emotional intelligence, cognitive intelligence, and cytokines. Medicine 98:e15345. doi: 10.1097/MD.0000000000015345, PMID: 31045776 PMC6504531

[ref33] Kang D. M. (2020). An elementary school EFL teacher’s emotional intelligence and emotional labor. J. Lang. Ident. Educ. 21, 1–14. doi: 10.1080/15348458.2020.1777867

[ref34] Karakus M. Toprak M. Caliskan O. Crawford M. (2024). Teachers’ affective and physical well-being: emotional intelligence, emotional labour and implications for leadership. Int. J. Educ. Manag. 38, 469–485. doi: 10.1108/ijem-07-2023-0335, PMID: 35579975

[ref35] Karatepe O. M. (2013). High-performance work practices and hotel employee performance: the mediation of work engagement. Int. J. Hosp. Manag. 32, 132–140. doi: 10.1016/j.ijhm.2012.05.003

[ref36] Karim J. Weisz R. (2011). Emotional intelligence as a moderator of affectivity/emotional labor and emotional labor/psychological distress relationships. Psychol. Stud. 56, 348–359. doi: 10.1007/s12646-011-0107-9

[ref37] Kim J. S. (2023). Effect of psychological meaningfulness on job involvement, proactive behavior, and performance: focusing on the mediating effect of self-efficacy. Sustain. For. 15:10208. doi: 10.3390/su151310208

[ref38] Kokkinos C. M. Voulgaridou I. (2024). Emotional intelligence across the personality spectrum: A study of university students' personality profiles. Personal. Individ. Differ. 222:112574. doi: 10.1016/j.paid.2024.112574

[ref39] Law K. S. Wong C. S. Song L. J. (2004). The construct and criterion validity of emotional intelligence and its potential utility for management studies. J. Appl. Psychol. 89, 483–496. doi: 10.1037/0021-9010.89.3.483, PMID: 15161407

[ref40] Lee Y. H. Chelladurai P. (2015). Affectivity, emotional labor, emotional exhaustion, and emotional intelligence in coaching. J. Appl. Sport Psychol. 28, 170–184. doi: 10.1080/10413200.2015.1092481, PMID: 39804493

[ref41] Lee Y. H. Chelladurai P. (2017). Emotional intelligence, emotional labor, coach burnout, job satisfaction, and turnover intention in sport leadership. Eur. Sport Manag. Q. 18, 393–412. doi: 10.1080/16184742.2017.1406971

[ref42] Lee L. Madera J. M. (2019). Faking it or feeling it: the emotional displays of surface and deep acting on stress and engagement. Int. J. Contemp. Hosp. Manag. 31, 1744–1762. doi: 10.1108/ijchm-05-2018-0405

[ref43] Lennard A. C. Scott B. A. Johnson R. E. (2019). Turning frowns (and smiles) upside down: A multilevel examination of surface acting positive and negative emotions on well-being. J. Appl. Psychol. 104, 1164–1180. doi: 10.1037/apl0000400, PMID: 30829510

[ref44] Levine Brown E. Vesely C. K. Mehta S. Stark K. (2022). Preschool teachers’ emotional acting and school-based interactions. Early Childhood Educ. J. 51, 615–626. doi: 10.1007/s10643-022-01326-1, PMID: 35233161 PMC8874102

[ref45] Li J. B. Deng J. Xu Y. Sun J. Chen J. Datu J. A. D.. (2024a). Which well-being elements are fundamental for early childhood educators in the Chinese context? A network analysis. Appl. Res. Qual. Life 19, 103–134. doi: 10.1007/s11482-023-10233-5

[ref46] Li J. B. Xu Y. Sun J. Qiu S. Zhang R. Yang A. (2024b). A multilevel latent profile analysis of job demands, job resources, and personal resources in early childhood education: implications for multidimensional well-being. J. Sch. Psychol. 109:101405. doi: 10.1016/j.jsp.2024.101405, PMID: 40180459

[ref47] Lim H. Lee T. J. Weng C. Lee S. M. (2024). Effects of surface acting on exhaustion of Korean school counselors. J. Couns. Dev. 103, 49–59. doi: 10.1002/jcad.12536, PMID: 39814823

[ref48] Liu Y. Prati L. Perrewé P. L. Ferris G. R. (2008). The relationship between emotional resources and emotional labor: an exploratory study. J. Appl. Soc. Psychol. 38, 2410–2439. doi: 10.1111/j.1559-1816.2008.00398.x

[ref49] Lu Q. Ishak N. A. (2022). Teacher’s emotional intelligence and employee brand-based equity: mediating role of teaching performance and teacher’s self-efficacy. Front. Psychol. 13:901019. doi: 10.3389/fpsyg.2022.901019, PMID: 35783736 PMC9249126

[ref50] McCray J. Joseph Richard P. (2020). Towards a model of resilience protection: factors influencing doctoral completion. High. Educ. 80, 679–699. doi: 10.1007/s10734-020-00507-4

[ref51] Mérida López S. Extremera N. (2020). The interplay of emotional intelligence abilities and work engagement on job and life satisfaction: which emotional abilities matter most for secondary-school teachers. Front. Psychol. 11:563634. doi: 10.3389/fpsyg.2020.563634, PMID: 33192836 PMC7606868

[ref52] Näring G. Briët M. Brouwers A. (2006). Beyond demand–control: emotional labour and symptoms of burnout in teachers. Work Stress 20, 303–315. doi: 10.1080/02678370601065182

[ref53] Pervaiz S. Ali A. Asif M. (2019). Emotional intelligence, emotional labor strategies and satisfaction of secondary teachers in Pakistan. Int. J. Educ. Manag. 33, 721–733. doi: 10.1108/ijem-12-2017-0350

[ref54] Phuoc N. H. Hau L. N. Thuy P. N. (2022). The dual outcomes of frontliner’s autonomous motivation and deep acting in service co-creation: a dyadic approach. Serv. Bus. 16, 159–186. doi: 10.1007/s11628-021-00473-6

[ref55] Prentice C. Chen P. J. King B. (2013). Employee performance outcomes and burnout following the presentation-of-self in customer-service contexts. Int. J. Hosp. Manag. 35, 225–236. doi: 10.1016/j.ijhm.2013.06.007

[ref56] Qi X. Ji S. Zhang J. Lu W. Sluiter J. K. Deng H. (2017). Correlation of emotional labor and cortisol concentration in hair among female kindergarten teachers. Int. Arch. Occup. Environ. Health 90, 117–122. doi: 10.1007/s00420-016-1179-6, PMID: 27804039

[ref57] Rucker D. D. Preacher K. J. Tormala Z. L. Petty R. E. (2011). Mediation analysis in social psychology: current practices and new recommendations. Soc. Personal. Psychol. Compass 5, 359–371. doi: 10.1111/j.1751-9004.2011.00355.x

[ref58] Salovey P. Mayer J. D. (1990). Emotional intelligence. Imagin. Cogn. Pers. 9, 185–211. doi: 10.2190/DUGG-P24E-52WK-6CDG

[ref59] Schaufeli W. B. Bakker A. B. Salanova M. (2006). The measurement of work engagement with a short questionnaire. Educ. Psychol. Meas. 66, 701–716. doi: 10.1177/0013164405282471

[ref60] Schaufeli W. B. Salanova M. Gonzálezromá V. Bakker A. B. (2002). The measurement of engagement and burnout: a two sample confirmatory factor analytic approach. J. Happiness Stud. 3, 71–92. doi: 10.1023/A:1015630930326

[ref61] Schwab S. (2019). Teachers’ student-specific self-efficacy in relation to teacher and student variables. Educ. Psychol. 39, 4–18. doi: 10.1080/01443410.2018.1516861

[ref62] Sciotto G. Pace F. (2022). The role of surface acting in the relationship between job stressors, general health and need for recovery based on the frequency of interactions at work. Int. J. Environ. Res. Public Health 19:4800. doi: 10.3390/ijerph19084800, PMID: 35457670 PMC9024759

[ref63] Sousan A. Farmanesh P. Zargar P. (2022). The effect of surface acting on job stress and cognitive weariness among healthcare workers during the COVID-19 pandemic: exploring the role of sense of community. Front. Psychol. 13:826156. doi: 10.3389/fpsyg.2022.826156, PMID: 35360579 PMC8961440

[ref64] Tan S. Y. Chou C. C. (2018). Supervision effects on self-efficacy, competency, and job involvement of school counsellors. J. Psychol. Couns. Sch. 28, 18–32. doi: 10.1017/jgc.2017.19

[ref65] Tang X. Gu Y. (2024). Influence of leaders’ emotional labor and its perceived appropriateness on employees’ emotional labor. Behav. Sci. 14:413. doi: 10.3390/bs14050413, PMID: 38785905 PMC11117495

[ref66] Tiwari V. Shukla J. (2023). A study of job involvement among teacher educators. Res. Rev. Int. J. Multidis. 8, 116–120. doi: 10.31305/rrijm.2023.v08.n09.016

[ref67] Tschannen Moran M. Hoy A. W. Hoy W. K. (1998). Teacher efficacy: its meaning and measure. Rev. Educ. Res. 68, 202–248. doi: 10.3102/00346543068002202

[ref68] Tschannen-Moran M. Hoy A. W. (2001). Teacher efficacy: capturing an elusive construct. Teach. Teach. Educ. 17, 783–805. doi: 10.1016/S0742-051X(01)00036-1

[ref69] Turin O. Davidson S. (2022). Riding the tiger: professional capital and the engagement of Israeli kindergarten teachers with parents' Whats app groups. J. Prof. Capital Commun. 7, 334–352. doi: 10.1108/JPCC-04-2022-0023

[ref70] Turner K. Stough C. (2020). Pre-service teachers and emotional intelligence: a scoping review. Aust. Educ. Res. 47, 283–305. doi: 10.1007/s13384-019-00352-0

[ref71] Udayar S. Fiori M. Bausseron E. (2020). Emotional intelligence and performance in a stressful task: the mediating role of self-efficacy. Personal. Individ. Differ. 156:109790. doi: 10.1016/j.paid.2019.109790, PMID: 39812234

[ref72] Wang H. Hall N. C. Taxer J. L. (2019). Antecedents and consequences of teachers’ emotional labor: a systematic review and meta-analytic investigation. Educ. Psychol. Rev. 31, 663–698. doi: 10.1007/s10648-019-09475-3

[ref73] Willoughby E. Boutwell B. B. (2018). Importance of intelligence and emotional intelligence for physicians. J. Am. Med. Assoc. 320:205. doi: 10.1001/jama.2018.6278, PMID: 29998331

[ref74] Wong C. S. Law K. S. (2002). The effects of leader and follower emotional intelligence on performance and attitude: an exploratory study. Leadership Quart 13, 243–274. doi: 10.1016/s1048-9843(02)00099-1

[ref75] Xu Y. Wang I. Chen J. Sun J. Li J. B. (2024). The associations between early childhood educators' social-emotional competence and a wide range of outcomes: A three-level meta-analysis. Learn. Individ. Differ. 114:102521. doi: 10.1016/j.lindif.2024.102521, PMID: 39812234

[ref76] Yan Z. Yu S. Lin W. (2023). Parents’ perceived social support and children’s mental health: the chain mediating role of parental marital quality and parent–child relationships. Curr. Psychol. 43, 4198–4210. doi: 10.1007/s12144-023-04625-x, PMID: 39808227

[ref77] Yin H. (2012). Adaptation and validation of the teacher emotional labour strategy scale in China. Educ. Psychol. 32, 451–465. doi: 10.1080/01443410.2012.674488

[ref78] Yin H. (2015). The effect of teachers’ emotional labour on teaching satisfaction: moderation of emotional intelligence. Teach. Teach. 21, 789–810. doi: 10.1080/13540602.2014.995482

[ref79] Yin H. Huang S. Chen G. (2019). The relationships between teachers’ emotional labor and their burnout and satisfaction: A meta-analytic review. Educ. Res. Rev. 28:100283. doi: 10.1016/j.edurev.2019.100283

[ref80] Yin H. Lee J. C. K. (2012). Be passionate, but be rational as well: emotional rules for Chinese teachers’ work. Teach. Teach. Educ. 28, 56–65. doi: 10.1016/j.tate.2011.08.005

[ref81] Yin H. Lee J. C. K. Zhang Z. Jin Y. (2013). Exploring the relationship among teachers' emotional intelligence, emotional labor strategies and teaching satisfaction. Teach. Teach. Educ. 35, 137–145. doi: 10.1016/j.tate.2013.06.006

[ref82] Zheng X. Shi X. Liu Y. (2020). Leading teachers’ emotions like parents: relationships between paternalistic leadership, emotional labor and teacher commitment in China. Front. Psychol. 11:00519. doi: 10.3389/fpsyg.2020.00519, PMID: 32318001 PMC7147470

[ref83] Zvobgo V. Abraham R. Sabharwal M. (2021). Faking versus feeling emotions: does personality–job fit make a difference. Public Pers. Manag. 51, 125–148. doi: 10.1177/00910260211034213, PMID: 39807426

